# Inducible Mtld expression facilitated the introduction of the mannitol synthesis pathway in *Synechococcus elongatus* PCC 7942

**DOI:** 10.3389/fbioe.2025.1575266

**Published:** 2025-03-21

**Authors:** Jiahui Sun, Jinyu Cui, Xuejing Xu, Jinhui Tang, Huili Sun, Xiangxiao Liu, Xiangyi Yuan, Guodong Luan, Xuefeng Lu

**Affiliations:** ^1^ Qingdao Institute of Bioenergy and Bioprocess Technology, Chinese Academy of Sciences, Qingdao, China; ^2^ Shandong Energy Institute, Qingdao, China; ^3^ Qingdao New Energy Shandong Laboratory, Qingdao, China; ^4^ College of Life Science, University of Chinese Academy of Sciences, Beijing, China; ^5^ Dalian National Laboratory for Clean Energy, Dalian, China; ^6^ Qingdao National Laboratory for Marine Science and Technology, Qingdao, China

**Keywords:** cyanobacteria, mannitol, *Synechococcus elongatus* PCC 7942, mannitol-1-phosphate dehydrogenase, mannitol-1-phosphatase

## Abstract

Mannitol is a valuable sugar alcohol, extensively used across various industries. Cyanobacteria show potential as future platforms for mannitol production, utilizing CO_2_ and solar energy directly. The proof-of-concept has been demonstrated by introducing a two-step pathway in cyanobacteria, converting fructose-6-phosphate to mannitol-1-phosphate and sequentially to mannitol. However, recombinant strains generally faced issues of genetic instability or low titers, consequently affecting the long-term mannitol production. In this work, the construction strategy for engineering mannitol production in *Synechococcus elongatus* PCC 7942, based on commonly adopted pathway comprising mannitol-1-phosphate dehydrogenase (Mtld) and mannitol-1-phosphatase (M1Pase), was optimized. The results demonstrated that the sequential introduction of *m1p* and *mtld* was required to obtain mannitol-producing strains. We further manipulated the abundances of Mtld with a theophylline dose-responsive riboswitch approach, and by combining it with the overexpression of *m1p*, we successfully obtained a recombinant strain producing 1.5 g/L mannitol under optimal conditions, the highest cyanobacterial yield to date. In addition, the controlled expression of *mtld* was demonstrated to remarkably augment the genetic stability of the mutant under long-term culturing circumstances, which continued to secrete mannitol after more than 2 months of cultivation without the addition of theophylline, and the mannitol biosynthesis operon did not undergo any spontaneous mutation. The findings in this work provided novel insights into the area of cyanobacteria mannitol metabolism engineering, and would inspire researchers to construct strains with different gene regulatory strategies for efficient photosynthetic biosynthesis.

## 1 Introduction

Mannitol, also known as d-mannitol, is a six-carbon sugar alcohol naturally found in fungi, bacteria, yeasts, and various plants, exhibiting stable chemical properties and unique sugar-like attributes such as low sweetness, low calories, and minimal hygroscopicity (Chen et al., 2020; Juszczyk et al., 2023; Yang et al., 2022). Traditionally, mannitol was derived from seaweed harvested during specific seasons, a method constrained by both low efficiency and ecological impacts (Xiao et al., 2024). Currently, the predominant technique for synthesizing mannitol involves the catalytic hydrogenation of a fructose/glucose mixture using nickel as a catalyst under elevated temperatures and pressures (Saha and Saha, 2007; Bhatt et al., 2013). However, this conventional approach faces challenges such as high energy consumption, environmental concerns, and the need for expensive purification processes (Rodiansono et al., 2019). Recent advances in microbial biotechnology have enabled the biosynthesis of mannitol through genetically engineered lactic acid bacteria expressing mannitol dehydrogenase (Mdh), which catalyzes the bioconversion of fructose under mild conditions (Zhang et al., 2018; Saha, 2004). This biological approach represents a sustainable alternative to conventional chemical synthesis methods, operating at ambient temperatures and neutral pH while minimizing energy consumption and hazardous byproducts (Dai et al., 2017). Despite its potential, microbial mannitol production faces challenges in scalability and sustainability due to heterotrophic microorganisms' dependence on organic carbon sources, leading to issues of resource competition, cost volatility, and geographical constraints (Bhatt et al., 2012; Martínez-Miranda et al., 2022).

**FIGURE 1:**
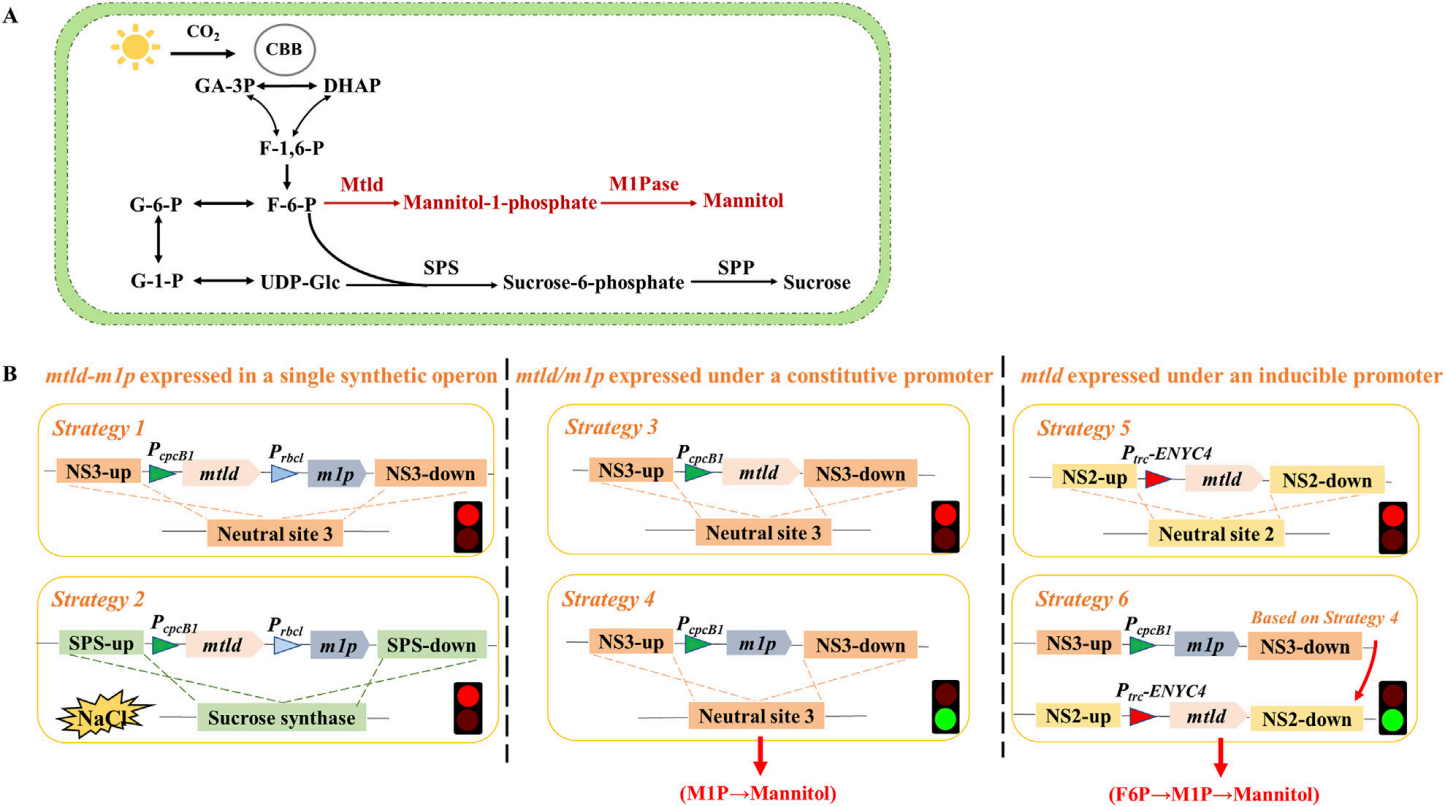
Metabolic pathway diagram of mannitol-producing strains and schematic diagram of strain construction process. **(A)** Schematic metabolic network of the engineered mannitol biosynthetic pathway in PCC 7942. Black lines represent reactions catalyzed by endogenous *Synechococcus* enzymes; whereas, the metabolic pathways marked in red are newly introduced pathway. Abbreviations: CBB cycle, Calvin-Benson-Bassham cycle; F-1,6-P, fructose-1,6-bisphosphate; F-6-P, fructose-6-phosphate; UDP-Glc, UDP-glucose; G-6-P, glucose-6-phosphate; G-1-P, glucose-1-phosphate; Sps, sucrose-phosphate synthase; Spp, sucrose-6-phosphatase; Mtld, mannitol-1-phosphate dehydrogenase; M1Pase, mannitol-1-phosphatase. The two-step conversion process initiated with the catalysis of mannitol-1-phosphate dehydrogenase (Mtld) converting fructose-6-phosphate (F6P) into mannitol-1-phosphate (M1P), followed by the hydrolysis of M1P into mannitol by mannitol-1-phosphatase (M1Pase). **(B)** The attempts to construct mannitol-producing strains. See Results Section for descriptions of specific strategies.

Cyanobacteria, as photosynthetic prokaryotes, have gained increasing attention as platforms for renewable chemical production due to their simple genetic background, rapid growth rate, and capability of direct CO_2_ fixation (Knoot et al., 2018; Zhang et al., 2024). Establishing cyanobacterial photosynthetic cell factories for the direct conversion of CO_2_ into mannitol represents a novel and promising route (Klähn et al., 2024; Cui et al., 2023). The introduction of a mannitol biosynthesis pathway consisting of two enzymes (Mtld and M1Pase) into the cyanobacterial genome enables the conversion of the Calvin cycle intermediate fructose-6-phosphate (F6P) to mannitol-1-phosphate (M1P), and ultimately to mannitol (Figure 1A) (Pritam et al., 2023). Multiple *Synechococcus* and *Synechocystis* strains have been genetically modified via the reconstruction of the two-step conversion process, and the engineered cell factories are capable of producing mannitol at concentrations between 0.1 and 1.1 g/L, with titers ranging from 2.8 to 91.7 mg/L/day (Table 1). Nevertheless, the *mtld-m1p* pathway exhibits instability and potential cytotoxicity in various cyanobacterial hosts. Long-term cultivations of the mannitol-producing strains resulted in a decrease in mannitol production due to spontaneous mutations within the mannitol biosynthesis operon, ultimately impacting the stability and sustainability of the synthesis process (Jacobsen and Frigaard, 2014; Wu et al., 2020). Against the backdrop, we aimed to develop more efficient and stable photosynthetic cell factories for mannitol production.

**TABLE 1:** Comparison of mannitol production in cyanobacteria determined in the present study with published values.

Cyanobacterial strain	Highest yield	Cultivation days	Characteristics of modified strain	Culture condition	References
PCC 7002	1.1 g/L	12	∆*desB::P*_*psbA*_ *-mtld-m1p* glycogen synthase deficient (∆*glgA1;* ∆*glgA2*)	30°C, 250 μmol m^−2^ s^−1^, 1% CO_2_	Jacobsen and Frigaard (2014)
PCC 7002	0.1 g/L	52	Algal Fusion protein M1PDH/M1Pase	30°C, 150 μmol m^–2^ s^–1^ with 16/8 h photoperiod, 0.04% CO_2_ with 300 mM NaCl	Madsen et al. (2018)
PCC 6803	0.018 g/L OD_730_	7	*Slr0168::P*_*trc*_ *-mtld-m1p*	30°C, 50 μmol m^−2^ s^−1^, 0.04% CO_2_ with NaCl induction	Wu et al. (2020)
PCC 11801	0.4 g/L	12	*NS1::P*_*rbc225*_ *-mtld-P*_*psbA1*_ *-m1p*	3 days: 38°C, 30 μmol m^–2^ s^–1^, 0.04% CO_2_ 9 days: 38°C, 30 μmol m^–2^ s^–1^, 1% CO_2_	Pritam et al. (2023)
PCC 11802	0.54 g/L	12	*NS1::P*_*rbc225*_ *-mtld-P*_*psbA1*_ *-m1p*	3 days: 38°C, 30 μmol m^–2^ s^–1^, 0.04% CO_2_ 9 days: 38°C, 30 μmol m^–2^ s^–1^, 1% CO_2_	Pritam et al. (2023)
PCC 7942	0.7 g/L	12	*NS1::P*_*cpcB300*_ *-mtld-P*_*psbA1*_ *-m1p*	3 days: 38°C, 300 μmol m^–2^ s^–1^, 0.04% CO_2_ 9 days: 38°C, 30 μmol m^–2^ s^–1^, 1% CO_2_	Pritam et al. (2023)
PCC 7942	1.5 g/L	12	NS2*::*P_*trc*_-*enyc4-mtld;* NS3*::*P_*cpcB*_ *-m1p*	30°C, 200 μmol m^−2^ s^−1^, 3% CO_2_, with a final theophylline concentration of 100 µM	This study

This work demonstrated multiple strain construction attempts to engineer *Synechococcus elongatus* PCC 7942 (hereafter PCC 7942 for short) for direct photosynthetic mannitol production based on commonly adopted pathway comprising *mtld* and *m1p*. Previous studies have utilized strong and constitutive promoters (for instance, *P*_*psbA*_) to co-express the *mtld-m1p* gene (Table 1). To ensure stable and efficient mannitol production, we manipulated the conventional mannitol metabolism pathway (F6P→M1P→Mannitol) by regulating the expression of *mtld* adopting a theophylline-dose responsive riboswitch system ENYC4 in PCC 7942, and further optimized the theophylline concentration to avoid genotypic mutations triggered by excessive M1P in strains. At the optimal theophylline concentration, the total mannitol titer exceeding 1.5 g/L were achieved. Compared to earlier efforts (Jacobsen and Frigaard, 2014), our work achieves a 36% higher mannitol titer (from 1.1 g/L to 1.5 g/L over the same incubation period) by synergizing inducible pathway regulation with carbon flux optimization. In addition, strain JS226 maintained its mannitol synthesis capability in the absence of theophylline, with no spontaneous mutations occurring in the mannitol biosynthesis operon over a period of more than 2 months. In summary, the optimized Mtld-M1P pathway notably improved the phenotypic stability of the engineered strains throughout the cultivation process and preliminary solved the genetically unstable issues of mutants in cyanobacteria metabolic engineering cases for mannitol producing. These results provide valuable insights into mannitol metabolism in cyanobacteria and shed light on the plasticity of cyanobacterial sugar metabolism networks.

## 2 Materials and methods

### 2.1 Chemicals and reagents

Unless noted, all reagents were purchased from Sigma-Aldrich (United States). The SeamLess Cloning Kit, 2×Taq PCR Master Mix and 2×Phanta Max Master Mix were purchased from Vazyme Biotech (Nanjing, China). Primer synthesis and DNA sequencing were carried out by TsingKe (Beijing, China).

### 2.2 Construction of plasmids and strains

*Escherichia coli* DH5α was used as the host for the construction of plasmids. *m1p* from *Eimeria tenella* (Allocco et al., 1999) were codon-optimized and synthesized by TsingKe (Beijing, China). *mtlD* was amplified from the genomic DNA of *E. coli* MG1655. The respective plasmids would be transformed into *Synechococcus* cells, and the antibiotic resistant transformants were usually obtained after 7–10 days of cultivation on selective BG11 agar plates. The genotypes of the transformants were verified by PCR and DNA-sequencing. The plasmids and strains constructed in this study are listed in Supplementary Tables S1, S2, respectively.

### 2.3 Cyanobacterial cultivation conditions

For standard cultivation, the wild-type and recombinant strains were grown in BG11 medium. Strains inoculated into 25 mL medium were precultured in a rotating shaking incubator (150 rpm) under continuous illumination (30°C, 30–50 μmol photons/m^2^/s). Then, each preculture was re-inoculated into 200 mL medium in a flask under continuous illumination and aeration until the culture reached the exponential growth period. Cells were resuspended with 65 mL fresh culture media to an initial OD_730_ of approximately 1.0 in column photobioreactors (with a diameter of 3 cm). When evaluated in a column photobioreactor, cyanobacterial strains were cultivated at 30°C under constant white-light illumination of 150–200 μmol photons/m^2^/s aerated with 3% (vol/vol) CO_2_-supplemented air. In addition, 20 μg/mL kanamycin, 10 μg/mL chloramphenicol, or 2 μg/mL gentamicin was added to the BG11 medium when needed. Theophylline will be added as needed from the beginning of cultivation (day 0) to induce mannitol production (Dan et al., 2022). Aliquots of cells were sampled for measuring cell growth and sugar production.

### 2.4 Detection method of mannitol

For the detection of extracellular mannitol, 1 mL of algal liquid of the strains was centrifuged at 13,000 rpm for 10 min, and then the centrifuged supernatant was transferred to another clean 1.5 mL EP tube. The extracellular mannitol extract was diluted and detected by a d-Mannitol Kit (K-MANOL, Megazyme). For detection of intracellular mannitol contents, the extraction method was as reported in previous literature (Qiao et al., 2020).

## 3 Results

### 3.1 Challenges in constructing co-expressing *mtld-m1p* strain in PCC 7942

Using PCC 7942 as the chassis for mannitol production, construction strategy with the conventional two-step pathway (F6P→M1P→Mannitol) was initially evaluated and optimized. The mannitol biosynthetic pathway (*mtlD-m1p*) was firstly organized in a single synthetic operon inserted into the neutral site 3 (plasmid pJS118) of PCC 7942 chromosome by homologous recombination (Figure 1B, Strategy 1). However, regardless of several attempts, no transformants with successful genomic integration of the *mtlD-m1p* operon could be obtained. Under salt stress, PCC 7942 synthesizes sucrose as a compatible solute to maintain osmotic balance and protect cellular structures (Ducat et al., 2012). Similarly, mannitol, another compatible solute, has also been shown to play essential roles in osmotic stress adaptation in cyanobacteria (Wu et al., 2020). Inspired by that, plasmid pJS123, was constructed containing the mannitol biosynthesis cassette inserted into the locus of sucrose synthase gene (*sps*) in the PCC 7942 chromosome, and applying salt stress as a selection pressure to facilitate the acquisition of transformants (Figure 1B, Strategy 2). However, the mutants could still not be obtained no matter on normal selective BG11 agar plates or plates supplemented with 50/150 mM NaCl. Thus, it was hypothesized that a complete mannitol biosynthesis operon (co-expression *mtlD* and *m1p*) may cause severe physiological and metabolic impairments in strains. Subsequently, plasmids pJS125 (Figure 1B, Strategy 3) and pJS126 (Figure 1B, Strategy 4) were designed to overexpress *mtlD*/*m1p* using a strong constitutive promoter at neutral site 3, respectively. While strains expressing the single *m1p* gene were easily segregated, efforts to construct strains containing *mtlD* failed for multiple times.

### 3.2 Sequential introduction of *m1p* and *mtlD* enables acquisition of mannitol-synthesizing strains

Since the strategies to construct strains containing *mtlD* gene under the control of a strong constitutive promoter failed to work, an inducible expression system was utilized for the expression of *mtlD*. Previously, the P_*trc-ENYC4*_ system (comprising P_*trc*_ promoter and the *ENYC4* riboswitch sequence) has been effectively utilized to regulate the expression of the critical genes of the glycogen synthesis and degradation (*glgC/glgP*) and facilitated flexible regulation of GlgC/GlgP abundances as well as glycogen contents (Dan et al., 2022; Chi et al., 2019). In this work, plasmids pJS166, integrating the P_*trc-ENYC4*_ element and *mtlD* expression cassette (Figure 1B, Strategy 5), was constructed and transformed into the strain JS201, resulting in the recombinant strain JS226 (WT-*m1p*-P_*trc-ENYC4*_ *-mtlD*) with successful genetic segregation in a single transformation process (Figure 1B, Strategy 6). It is worth mentioning that, despite several attempts to introduce plasmid pJS166 into the wild type strain (WT 7942), only one colony was obtained with a mutation in the 360^nd^ amino acid (Glutamine→Stop Codon), resulting in a premature termination codon (Supplementary Figure S1). Based on the construction process, it could be postulated that the principal obstacle in incorporating the *mtld-m1p* pathway into PCC 7942 stemmed from Mtld, as the catalytic reaction carried by Mtld or the resultant metabolites might potentially exert toxicity on cells.

Phosphate sugar is known to be toxic to bacterial cells. In *Corynebacterium glutamicum*, knocking out the gene *scrB* responsible for hydrolyzing sucrose-6-phosphate (Suc-6-P) leads to the accumulation of a significant amount of Suc-6-P, which impairs the growth capabilities of mutants (Engels et al., 2008). Mutants lacking fructose-1-phosphate kinase in *E. coli* are unable to utilize fructose-1-phosphate as a substrate, resulting in the accumulation of this toxic metabolite that damages cellular growth (Ferenci and Kornberg, 1973). Mannitol-1-phosphate has been reported to be a toxic intermediary affecting cellular functions (Sand et al., 2015). The addition of external mannitol significantly impeded the growth of *Salmonella typhimurium* mutants with the *mtlD* deletion (Berkowitz, 1971). Previous studies have reported the substantial leaky expression effect of the theophylline-responsive riboswitch (Dan et al., 2022; Chi et al., 2019). In our cases, the expression of *mtld* led to an increase in the intracellular concentration of M1P. This elevation was detrimental to *Synechococcus* cells and impeded the acquisition of the designed transformants. Such effects could be mitigated by the introduction of M1Pase, which was capable of converting the accumulated M1P, thereby rescuing the cells. Consequently, the sequential introduction of *m1p* and *mtld* was essential for obtaining mannitol-producing strains (Supplementary Figure S2).

### 3.3 High-efficiency production of mannitol by theophylline-dose regulating the expression of *mtlD* combining with continuous overexpression of *m1p*

**FIGURE 2:**
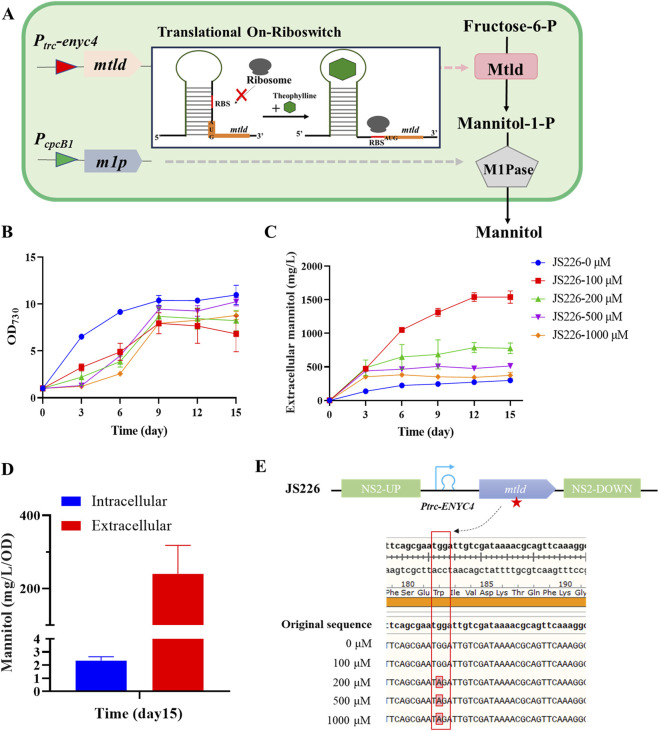
Engineering cyanobacteria to produce mannitol via sequential enzymatic reactions. **(A)** Schematic diagram of the heterologous overexpression of mtld/m1p in PCC 7942. Growth **(B)** and extracellular mannitol **(C)** of JS226 with a concentration of theophylline (0, 100, 200, 500, and 1000 μM) supplemented into the culture broth. **(D)** Extracellular/intracellular mannitol content of strain JS226 cultured in the column photobioreactor at day 15. Data are from representative experiments where error bars represent standard deviations from ≥3 replicates. **(E)** Genotype sequencing results of strain JS226cultured in the column photobioreactor at day 15. A single-base mutation had occurred in codon 182 of the mtlD gene and turned to stop codon at 200, 500 and 1000 μM theophylline concentrations, thus caused the loss of mannitol production capacity in engineered strains carrying mtlD-m1p pathway.

To evaluate the performance of recombinant strain for mannitol synthesis (Figure 2A), JS226 strain was cultivated with theophylline concentrations ranging from 0 to 1,000 µM to induce the expression of *mtld*. The added theophylline caused significantly growth reduction compared to the control (Figure 2B), whereas it had a negligible influence on that of WT 7942 (Supplementary Figure S3), indicating that the induced expression of *mtld* and production of mannitol had an adverse effect on the growth of the *Synechococcus* cells. This is consistent with previous studies that, PCC 7002 strains carrying the complete mannitol biosynthesis pathway exhibited severe growth impairment, displaying a chlorosis-like yellow-brown coloration, possibly due to the combined effect of physiological damage and metabolic rebalance in cells (Jacobsen and Frigaard, 2014). Mannitol biosynthesis exhibited optimal productivity during the initial cultivation phase, reaching a maximum production rate of 0.18 g/L/day within the first 6 days under 100 µM theophylline induction, ultimately achieving a cumulative mannitol concentration of 1.5 g/L by day 12 (Figure 2C). Intracellular mannitol contents were quantified on day 15 (Figure 2D), indicating that approximately 98% of mannitol was released into the culture medium. Additionally, even without theophylline supplementation, extracellular mannitol (approximately 0.3 g/L) was detectable due to leaky riboswitch expression. F6P, a significant metabolite of the Calvin Cycle, exhibits a robust metabolic flow particularly under photoautotrophic conditions, which may promote the conversion to mannitol even with minimal MtlD abundances.

### 3.4 Mutations in the *mtld* gene led to loss of mannitol production capacity in engineered strains

Since theophylline concentration above 200 µM did not enhance mannitol production, the integrity of the *mtld*/*m1p* element on chromosome of the recombinant strain cultivated with/without theophylline induction were checked through DNA sequencing. It was observed that, strains incubated without adding theophylline, or with the addition of 100 μM theophylline showed no mutations in the *mtld*/*m1p* operon. In contrast, the reduced mannitol productions in various subcultures at theophylline concentrations of 200, 500, and 1,000 µM were predominantly due to spontaneous mutations within the mannitol biosynthesis operon (Figure 2E). Specifically, a single-base mutation occurred in the 182^nd^ amino acid (Tryptophan→Stop codon). This nonsense mutation leads to the production of a truncated protein comprising only 182 amino acids, representing approximately 47.5% (182/383) of the complete polypeptide chain. Genetic instability has been observed in transgenic PCC 7942 strains producing ethylene by expressing an ethylene-forming enzyme (Takahama et al., 2003; Wang et al., 2021). The ethylene-producing strains exhibited chlorosis and impaired growth. However, with the cessation of ethylene production triggered by genetic instability, chlorosis was abolished and growth rates reverted to having growth rates similar to that of the WT levels. This phenomenon was very similar to observations on cell growth and mannitol production in JS226 during later stages of cultivation in this work (Figures 2B, C; Supplementary Figure S4). As previously mentioned, accumulated M1P might bring in cytotoxic effects, and the incorporation of M1Pase could alleviate the physiological stress. Thus, it could be hypothesized that the addition of high concentrations of theophylline would lead to an over-accumulation of M1P and an insufficient conversion of M1P by M1Pase, ultimately prompting mutations in the *mtld* gene as a stress-adaptive mechanism to alleviate physiological burden of strains.

**FIGURE 3:**
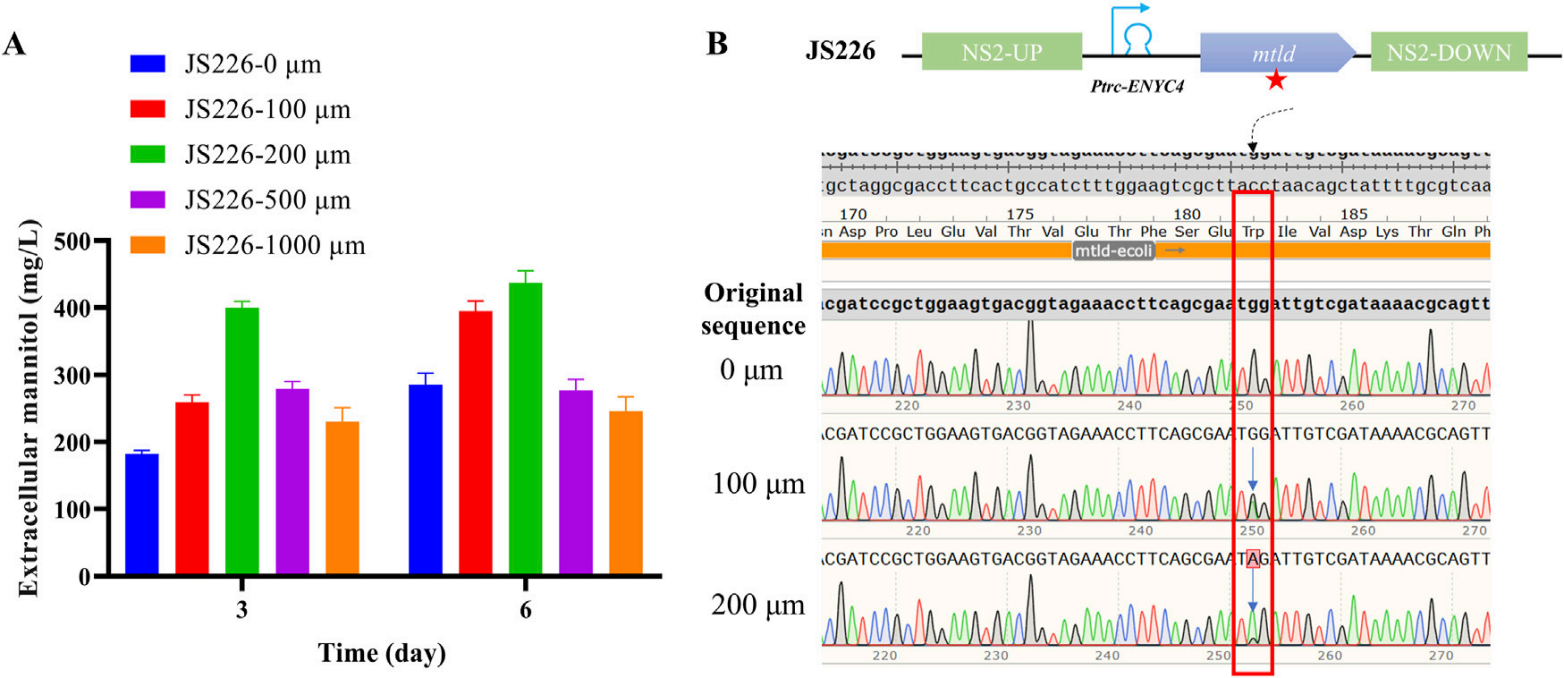
Re-evaluation of JS226 strain sub-cultured for 2 months and the DNA sequencing results of subcultures. **(A)** Extracellular mannitol content and **(B)** genotype sequencing results of strain JS226. For the subculture in a shaker table, the initial OD_730_ of each passage is set at 0.1, once the cell density reaches an OD_730_ of 2.5–3.5, an appropriate volume of cells is transferred to a fresh BG11 medium with initial OD_730_ at 0.1. Cultivation in the column photobioreactor was conducted at 30°C, 200 µmol photons/m^2^/s, cultured with 1 × BG11, and bubbled with 3% CO_2_.

Additionally, an increase in the passaging time of the JS226 strain led to significant fluctuations in mannitol productivity (Figures 3A, B). A re-evaluation of the JS226 strain following 2 months of subculturing disclosed that the *mtld* CDS (Coding DNA Sequence) had mutated in broths supplemented with 100, 200, 500, and 1,000 µM theophylline concentrations. Nevertheless, in the absence of theophylline, strain JS226 exhibited stable heritability and sustained mannitol secretion (0.3 g/L in 6 days), without any spontaneous mutations in the mannitol biosynthetic operon (Supplementary Figure S5; Figure 3B).

## 4 Discussion

Cyanobacteria are often engineered to synthesize substances with potential toxicity or physiological inhibitory effects, causing genetic instability in strains (Takahama et al., 2003; Wang et al., 2021). In this study, we improved the strain construction strategy to overcome these challenges. By using a theophylline dose-responsive riboswitch to regulate Mtld abundances and overexpressing M1Pase, the recombinant strain synthesized 1.5 g/L mannitol within 12 days, the highest mannitol yield of cyanobacteria in a single batch cultivation. More importantly, the recombinant showed good genetic stability in prolonged cultivation, indicating the risk of spontaneous mutations within the mannitol biosynthesis operon was effectively mitigated, thereby preventing a decline or cessation of mannitol production during the cultivation process.

Our findings suggest that previous failures in establishing stable cyanobacterial strains for the “two-step” mannitol pathway may be attributed to inadequate M1Pase-mediated conversion of M1P, leading to its intracellular accumulation and subsequent mutagenic effects on the *mtlD* gene. This hypothesis needs further investigation through quantitative analysis of intracellular M1P concentrations. Experiments could also be carried out to further advance cyanobacterial metabolic engineering for mannitol production: (i) engineering M1Pase variants through directed evolution to improve its catalytic efficiency and substrate specificity; (ii) exploring alternative routes for mannitol production, such as using fructose as a direct substrate for mannitol synthesis via the expression of a heterologous mannitol dehydrogenase (Mdh), which would eliminate the metabolic bottleneck associated with M1P accumulation and reducing metabolic burden on strains. These approaches may be crucial for stabilizing mannitol production and addressing genetic instability issues.

## Data Availability

The original contributions presented in the study are included in the article/Supplementary Material, further inquiries can be directed to the corresponding authors.
