# Supplementary material for: Inducible Mtld expression facilitated the introduction of the mannitol synthesis pathway in Synechococcus elongatus PCC 7942
Source: Front Bioeng Biotechnol. 2025 Mar 21;13:1575266. doi: 10.3389/fbioe.2025.1575266 (PMC11968706; doi:10.3389/fbioe.2025.1575266)
Supplement: Supplementary file 1 [file DataSheet1.DOCX]

Supplementary Material

# Supplementary Figures and Tables

## Supplementary Figures


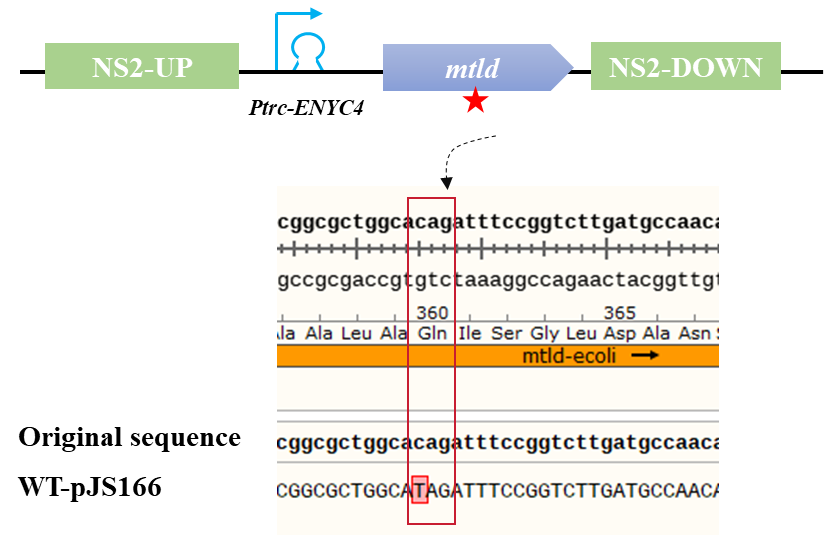


**Supplementary Figure 1.** **Attempt to introduce plasmid pJS166 (using theophylline-regulated promoter to drive the expression of *mtlD*) into WT 7942.** The 360^nd^ amino acid of *mtlD* gene was mutated to a stop codon (full length 383 amino acids).


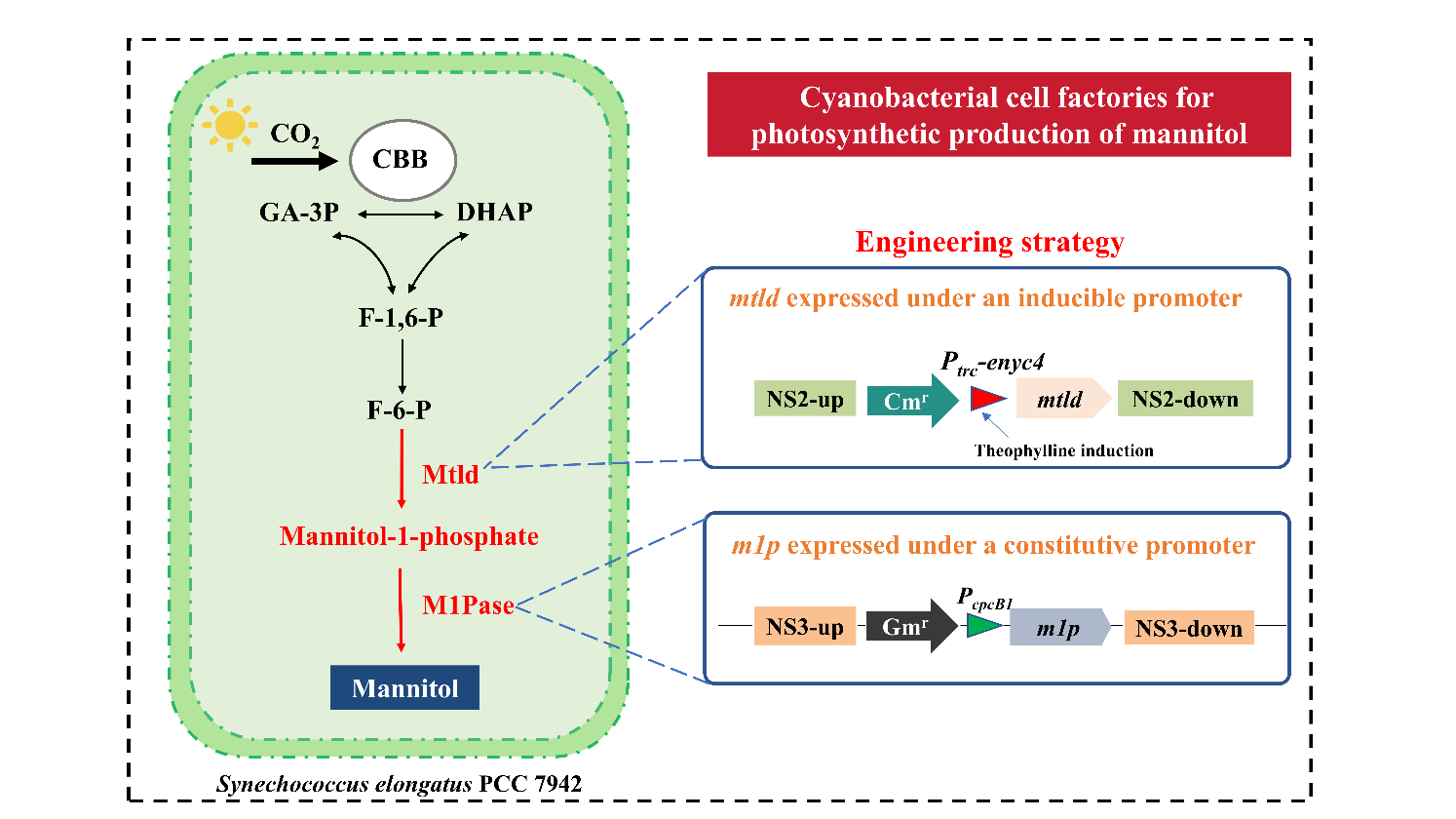


**Supplementary Figure 2. Metabolic pathway diagram of mannitol-producing strain JS226.** Gene integration loci: *mtlD* (mannitol-1-phosphate dehydrogenase) under theophylline-inducible promoter P*_trc-ENYC4_* at NS2; *m1p* (mannitol-1-phosphatase) under constitutive promoter P*_cpcB1_* at NS3. NS2 and NS3, different neutral sites in the genome of PCC 7942.

**Supplementary Figure 3.** **Growth curves of WT 7942 under different theophylline addition concentrations were observed.** To assess the impact of theophylline on cellular physiology, WT 7942 was inoculated with or without theophylline in column photobioreactors, and it was revealed that the addition of 1 mM theophylline caused negligible influence on the growth. Cultivation was conducted at 30℃, 200 µmol photons/m^2^/s, cultured with 1×BG11 and bubbled with 3% CO_2_.


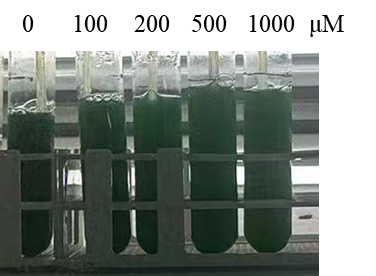


**Supplementary Figure 4. Coloration and growth recovery of mannitol-accumulating cyanobacterial cultures.** Phenotypic changes of cyanobacteria cultures under different theophylline concentrations after 12 days of cultivation (Figure 2B).


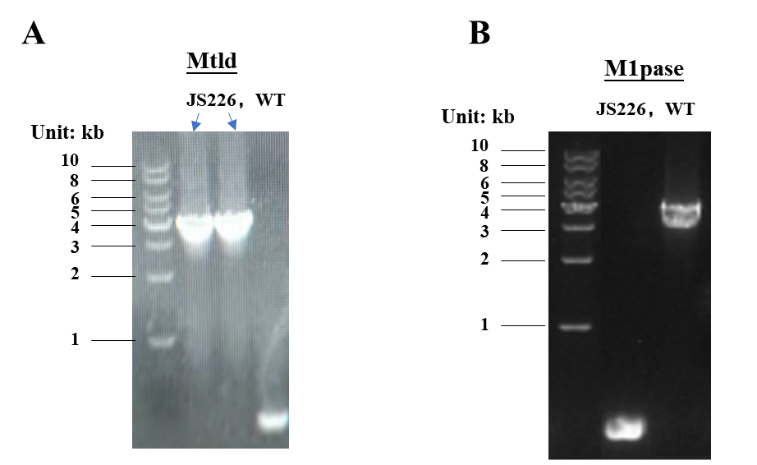


**Supplementary Figure 5. Genotype identification of the engineered strain JS226 and the wild-type (WT) control.** Primers specific to the neutral sites NS2 and NS3 were used to amplify the edited regions. (Expected band sizes: NS2, 3.8 kb; NS3: 3.3 kb).

## Supplementary Tables

**Supplementary Table 1.** Plasmids used in this study

| Plasmids | Relevant characteristics^a^ |
| --- | --- |
| pJS118 | NS3::P*_cpcB1_*-*mtld*-P*_rbcl_-m1p*-Km^r^ |
| pJS123 | Δ*sps_7942_*::P*_cpcB1_*-*mtld*-P*_rbcl_-m1p*-Km^r^ |
| pJS125 | NS3::P*_cpcB1_*-*mtld*-Km^r^ |
| pJS126 | NS3::P*_cpcB1_*-*m1p*-Gm^r^ |
| pJS166 | NS2::P*_trc_*-*enyc4*-*mtld-*Cm^r^ |

*^a^* NS2 and NS3, different neutral sites in the genome of PCC 7942; Cm^r^, chloramphenicol-resistance; Gm^r^, gentamycin-resistance; Km^r^, kanamycin-resistance.

**Supplementary Table 2.**

Strains used in this study

| Strains | Characteristics |
| --- | --- |
| JS201 | NS3::P*_cpcB_*-*m1p* |
| JS226 | JS201*-*NS2::P*_trc_*-*enyc4*-*mtld* |
